# Comparative Effectiveness of Single Versus Dual Adjuvant Therapy After Breast-Conserving Surgery in Hormone Receptor-Positive Ductal Carcinoma In Situ: A Multicenter Retrospective Cohort Study

**DOI:** 10.3390/jcm15218729

**Published:** 2026-11-09

**Authors:** Yuqiang Huang, Xianqiang Du, Junjing Li, Debo Chen

**Affiliations:** Department of Breast Surgery, Quanzhou First Hospital Affiliated to Fujian Medical University, Quanzhou 362000, China

**Keywords:** ductal carcinoma in situ, de-escalation, radiotherapy, endocrine therapy

## Abstract

**Background/Objectives:** To evaluate the comparative effectiveness of single versus dual adjuvant treatment strategies for hormone receptor-positive (HR+) ductal carcinoma in situ (DCIS) following breast-conserving surgery (BCS). **Methods:** This was a multicenter, retrospective study. We retrospectively identified 645 patients with HR+ DCIS who underwent BCS between 2009 and 2020 from the Shanghai Jiao Tong University Breast Cancer Database (SJTU-BCDB). Patients were stratified according to adjuvant treatment regimens: BCS alone, BCS combined with radiotherapy (BCS + RT), BCS combined with endocrine therapy (BCS + ET), and BCS combined with radiotherapy and endocrine therapy (BCS + RT + ET). The primary endpoint was recurrence-free survival (RFS). Multivariable Cox regression and inverse probability of treatment weighting (IPTW) analyses were performed to evaluate treatment associations with RFS outcomes. **Results:** The median follow-up was 74.0 months. In multivariable Cox regression, compared with BCS + RT + ET, neither BCS + RT (HR, 0.62; 95% CI, 0.12–3.13; *p* = 0.564) nor BCS + ET (HR, 1.49; 95% CI, 0.43–5.11; *p* = 0.525) showed a statistically significant difference in RFS. IPTW-adjusted analyses similarly showed no statistically significant differences between BCS + RT and BCS + RT + ET (HR, 0.28; 95% CI, 0.06–1.44; *p* = 0.128) or between BCS + ET and BCS + RT + ET (HR, 1.82; 95% CI, 0.49–6.82; *p* = 0.373). **Conclusions:** Among patients with HR+ DCIS undergoing BCS, no statistically significant difference in RFS was observed between dual-modality and single-modality adjuvant therapy. However, the limited number of events and wide confidence intervals limit the precision of estimates regarding the incremental benefit of combined RT and ET. Larger prospective studies with adequate statistical power are warranted to further define the comparative effectiveness of these treatment strategies.

## 1. Introduction

With the promotion of breast cancer (BC) screening and widespread use of mammography, the detection rate of ductal carcinoma in situ (DCIS) has substantially increased, accounting for approximately 20–25% of all newly diagnosed BC cases [1,2]. However, this increase in the diagnosis of DCIS has not been accompanied by an anticipated reduction in the incidence of invasive breast cancer, suggesting that a proportion of DCIS diagnoses may represent overdiagnosis [3,4]. Moreover, DCIS exhibits considerable biological heterogeneity, encompassing low-grade, indolent lesions and high-risk subtypes with substantial invasive potential, which poses significant challenges for establishing standardized treatment strategies [5]. Therefore, accurate identification of patients with different recurrence risks through refined risk stratification and the development of individualized management strategies has become a central priority in contemporary DCIS research and clinical practice.

Conventional management of DCIS, as established by pivotal clinical trials, such as NSABP B-17 and B-24, typically consists of breast-conserving surgery (BCS) followed by adjuvant radiotherapy (RT), with adjuvant endocrine therapy (ET) recommended for patients with hormone receptor-positive (HR+) DCIS [6]. Although these strategies have substantially reduced the risk of local recurrence (LRR), their widespread implementation has raised concerns about potential overtreatment, including therapy-related toxicities, psychological distress, and inefficient utilization of healthcare resources [4]. Consequently, increasing efforts have focused on evaluating treatment de-escalation strategies to determine whether selected patients may achieve acceptable oncological outcomes with less intensive treatment approaches while balancing recurrence prevention with treatment-related considerations [7,8,9]. In particular, therapeutic decision-making for HR+ DCIS remains challenging because of its dual sensitivity to both RT and ET. Although robust evidence supports the individual efficacy of RT and ET in reducing recurrence risk, the comparative effectiveness and clinical value of single versus combined dual adjuvant therapies (RT + ET), particularly in real-world HR+ DCIS populations, remain inadequately characterized [10]. This evidence gap contributes to ongoing clinical uncertainty regarding the optimal intensity of adjuvant treatment and represents an important barrier to individualized management of HR+ DCIS.

Accordingly, we established the first large-scale real-world cohort of Chinese patients with HR+ DCIS who underwent BCS. This study was designed to comprehensively evaluate the clinical effectiveness of single versus combined dual adjuvant therapies (RT + ET) following BCS in patients with HR+ DCIS and to provide real-world evidence regarding individualized adjuvant treatment strategies. We aimed to generate evidence that may inform clinical decision-making for HR+ DCIS by balancing potential oncologic benefits with treatment-related considerations, thereby contributing to more tailored approaches in DCIS management.

## 2. Materials and Methods

### 2.1. Patients and Study Design

This was a multicenter, retrospective observational cohort study. Patient baseline clinical characteristics were extracted from the Shanghai Jiao Tong University Breast Cancer Database (SJTU-BCDB), which included anonymized clinical data from 42 breast cancer centers across China. Among these centers, 37 contributed eligible patients included in the present analysis. Clinical and follow-up data of patients with HR+ DCIS who underwent BCS between January 2009 and December 2020 were retrospectively collected from the SJTU-BCDB. The inclusion criteria were as follows: (1) newly diagnosed, histologically confirmed pure DCIS, (2) status post-BCS, (3) age ≥ 18, and (4) pathological confirmation of estrogen receptor (ER)-positive and/or progesterone receptor (PR)-positive disease. The exclusion criteria were as follows: (1) male breast cancer, (2) bilateral breast cancer, (3) previous history of breast cancer or other malignant tumors, (4) lack of follow-up information, and (5) insufficient clinicopathological or treatment-related information required for the analysis.

### 2.2. Clinical Information Collection and Treatment Strategies

Patients were enrolled according to the aforementioned inclusion and exclusion criteria, and all participants underwent preoperative breast ultrasonography and mammography. Demographic and clinicopathological characteristics were collected from medical records and follow-up documentation, including age at diagnosis, menopausal status, tumor size, histological grade, Ki-67 expression, multifocality, clinical manifestation, ER and PR expression status, adjuvant treatment strategies, and follow-up outcomes. Tumor size was defined as the largest pathological dimension of the DCIS lesion measured in the surgical specimen after breast-conserving surgery. Imaging-detected mass size was not used as the measurement of tumor size. Molecular subtype classification was not routinely assigned because complete HER2 information was not consistently available across participating centers. Preoperative breast ultrasonography and mammography findings, including BI-RADS categories, were collected when available. Breast MRI was not routinely performed for all patients and was selectively performed according to institutional practice and clinical indications. Detailed MRI information was not consistently available across participating centers. Clinical presentation was categorized as mass-related or non-mass-related. Mass-related lesions were defined as palpable abnormalities detected by physical examination or measurable masses identified by imaging examination, whereas non-mass-related lesions included nipple discharge or mammographic abnormalities such as microcalcifications. Recurrence-free survival (RFS) was defined as the time from the date of surgery to the first occurrence of ipsilateral breast recurrence, contralateral breast cancer, regional recurrence, distant recurrence, or death from any cause. All patients underwent BCS and had histologically confirmed negative surgical margins (>2 mm). ER and PR expression in surgical specimens was routinely assessed by immunohistochemistry (IHC). HR+ DCIS was defined as ER-positive and/or PR-positive disease, with positivity defined as nuclear staining in ≥1% of tumor cells. Nuclear grade was classified as well differentiated (Grade I), moderately differentiated (Grade II), or poorly differentiated (Grade III). Patients were classified into four treatment groups according to the postoperative adjuvant treatment regimens actually received after breast-conserving surgery and pathological confirmation of DCIS: BCS alone, BCS + RT, BCS + ET, BCS + RT + ET. Endocrine therapy included tamoxifen, letrozole, anastrozole, and exemestane. Because the primary aim was to compare treatment strategies rather than individual endocrine therapy regimens, endocrine therapy was analyzed as a binary treatment exposure. Detailed treatment exposure information, including radiotherapy parameters and endocrine therapy duration or adherence, was not systematically available in this retrospective multicenter database.

### 2.3. Statistical Analysis

All statistical analyses were performed using R software version 4.6.0 (R Foundation for Statistical Computing, Vienna, Austria). Continuous variables are presented as medians with interquartile ranges (IQRs), and categorical variables are summarized as numbers and percentages. Comparisons of categorical variables were performed using the chi-squared test or Fisher’s exact test.

RFS was estimated using the Kaplan–Meier method, and differences among treatment groups were assessed using the log-rank test. Cox proportional hazard regression models were used to evaluate the association between postoperative adjuvant treatment strategies and RFS outcomes. Given the limited number of RFS events, multivariable Cox models were restricted to clinically relevant covariates, including age and tumor size, to minimize the risk of model overfitting. Hazard ratios (HRs) and corresponding 95% confidence intervals (CIs) are reported.

Because postoperative treatment allocation was determined by clinical decision-making rather than randomization, inverse probability of treatment weighting (IPTW) was performed to reduce potential confounding due to baseline differences between treatment groups. Propensity scores were estimated separately for the two prespecified pairwise comparisons using multivariable logistic regression models incorporating age, tumor size, histological grade, Ki-67 labeling index, ER expression, PR expression, and multifocality. Stabilized inverse probabilities of treatment weights targeting the average treatment effect (ATE) were calculated. The distribution of stabilized weights was examined using summary statistics, including the mean, standard deviation, minimum, 1st percentile, median, 99th percentile, and maximum. No weight truncation was applied in the primary analysis because no excessively large weights were observed. Effective sample size (ESS) after weighting was calculated as (Σw_i_)^2^/Σw_i_^2^. Covariate balance after weighting was evaluated using absolute standardized mean differences (SMDs), with values < 0.10 considered indicative of adequate balance. Propensity-score overlap and common support were assessed by visual inspection of the propensity-score distributions before and after weighting. Given the multicenter nature of this study and the long study period, additional sensitivity analyses were performed by further adjusting IPTW-weighted Cox regression models for diagnosis year as a continuous variable and center stratification to assess the robustness of the treatment effect estimates. Treatment effects after weighting were estimated using IPTW-weighted Cox proportional hazards models, with robust sandwich variance estimators used to obtain standard errors, 95% confidence intervals, and *p* values. IPTW-adjusted analyses were conducted for two prespecified pairwise comparisons: BCS + ET versus BCS + RT + ET and BCS + RT versus BCS + RT + ET. A two-sided *p* value < 0.05 was considered statistically significant.

## 3. Results

### 3.1. Patient and Clinicopathological Characteristics

**Figure 1:**
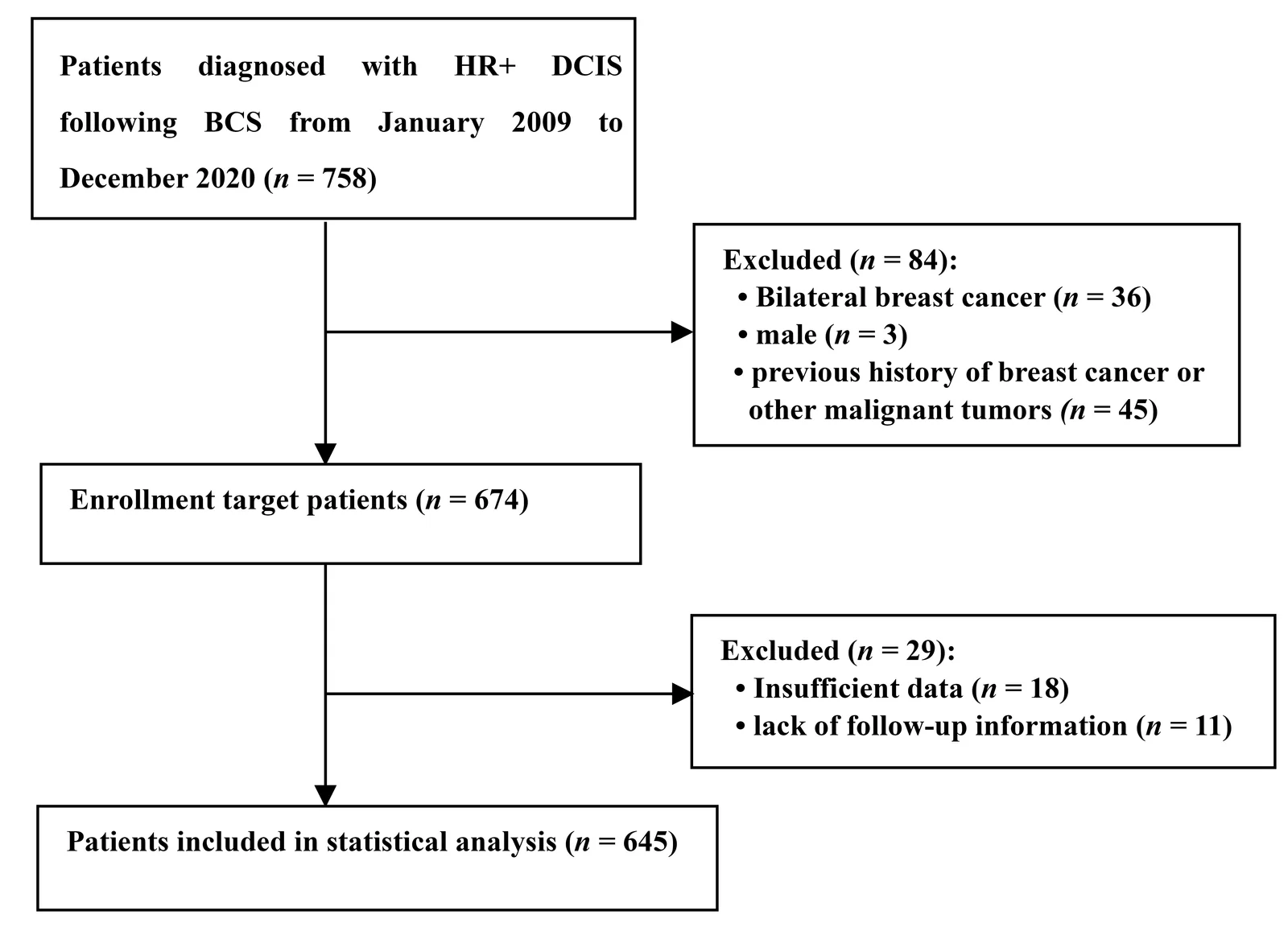
Flow diagram of the patient population. HR+, hormone receptor-positive; DCIS, ductal carcinoma in situ; BCS, breast-conserving surgery.

In this study, 758 patients with HR+ DCIS who underwent BCS were initially identified, and 645 patients with complete clinicopathological and follow-up data were included in the final analysis (Figure 1). The baseline demographic and clinicopathological characteristics of the study cohort are summarized in Table 1. The median follow-up duration was 74.0 months (IQR, 57.0–99.0 months). The median age at diagnosis was 48.0 years (IQR, 42.0–57.0 years), and 60.9% of patients were premenopausal. Most patients presented with unifocal disease (88.8%) and tumors ≤ 2 cm (71.6%). The majority of patients were aged ≤50 years (59.2%), exhibited low-to-intermediate histologic grade (grade I–II, 77.1%), demonstrated Ki-67 expression < 15% (62.8%), and presented with mass-related lesions (79.8%). ER expression > 10% and PR expression > 10% were documented in 88.7% and 74.1% of patients, respectively.

Of the four BCS-based initial treatment regimens, 304 patients (47.1%) received BCS + RT + ET, 136 (21.1%) received BCS + RT, 145 (22.5%) received BCS + ET, and 60 (9.3%) underwent BCS alone. Significant differences among the four treatment groups were observed in tumor size, ER expression, and PR expression (*p* = 0.009, *p* < 0.001, and *p* = 0.004, respectively). Specifically, the BCS + RT + ET group had the highest proportions of patients with tumors ≤ 20 mm (78.0%), ER expression > 10% (96.1%), and PR expression > 10% (78.3%). Significant differences were also observed in age distribution and menopausal status among treatment groups (*p* < 0.001 and *p* = 0.015, respectively), with relatively higher proportions of younger and premenopausal patients observed in the BCS + RT group.

### 3.2. RFS Outcomes According to Adjuvant Treatment Strategies

**Figure 2:**
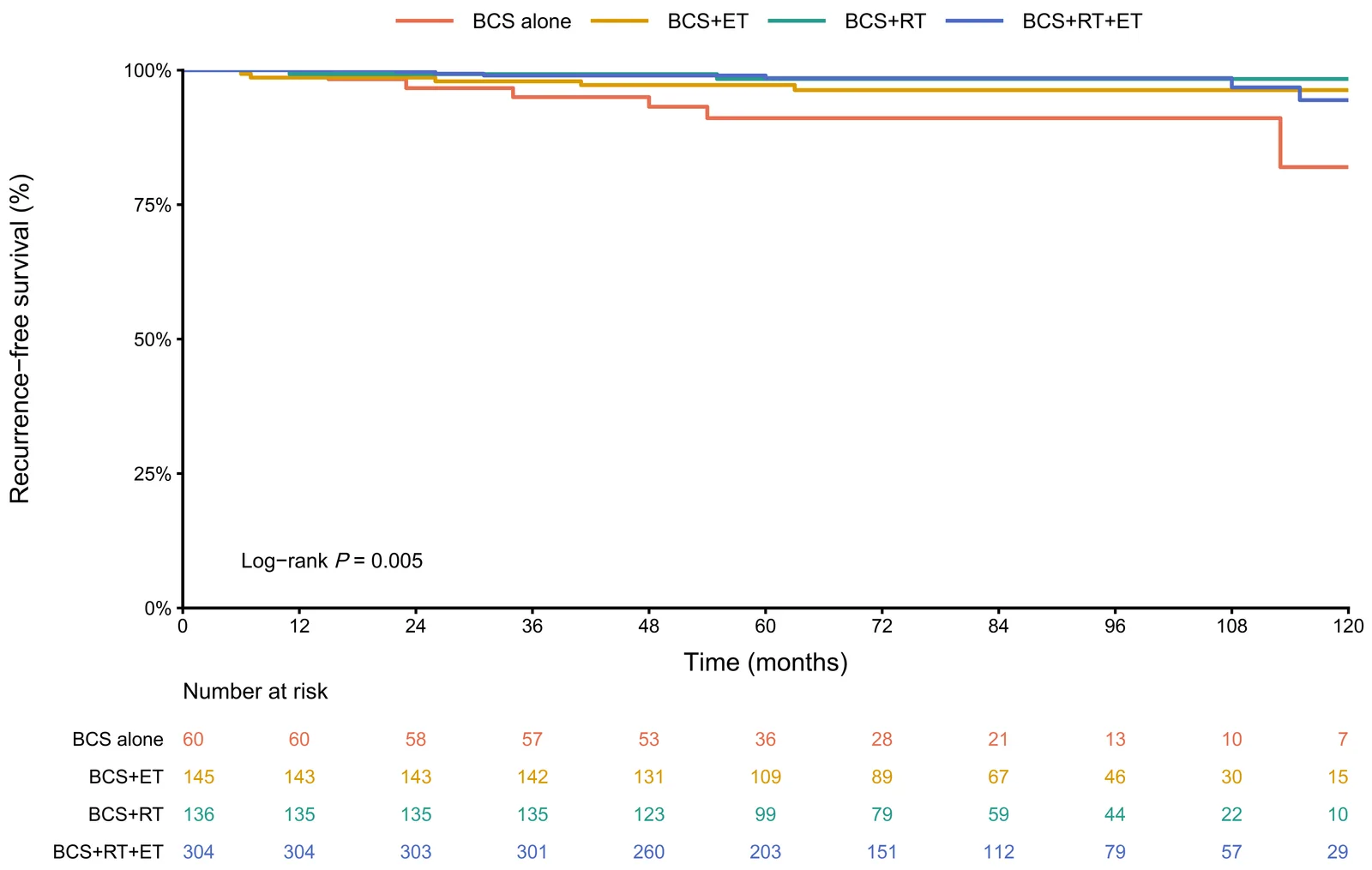
Kaplan–Meier survival curves according to adjuvant treatment strategies after breast-conserving surgery. BCS, breast-conserving surgery; RT, radiotherapy; ET, endocrine therapy.

During follow-up, 19 RFS events were documented among the 645 patients. Events occurred in 6 patients (10.0%) in the BCS-alone group, 2 patients (1.5%) in the BCS + RT group, 5 patients (3.4%) in the BCS + ET group, and 6 patients (2.0%) in the BCS + RT + ET group. Among the 19 first RFS events, 7 were ipsilateral breast events, including 1 invasive recurrence and 6 DCIS recurrences; 7 were contralateral breast events, including 2 invasive cancers and 5 DCIS events; 2 were distant recurrences; and 3 were deaths without recurrence. No regional recurrences were observed. The distribution of first RFS events according to treatment group is summarized in Supplementary Table S3. Kaplan–Meier analysis demonstrated a significant overall difference in RFS among the four treatment groups (log-rank *p* = 0.005) (Figure 2). Patients undergoing BCS alone experienced more RFS events than those receiving adjuvant therapy; however, this comparison should be interpreted cautiously given the small size of the BCS-alone group and the potential for confounding by indication.

**Figure 3:**
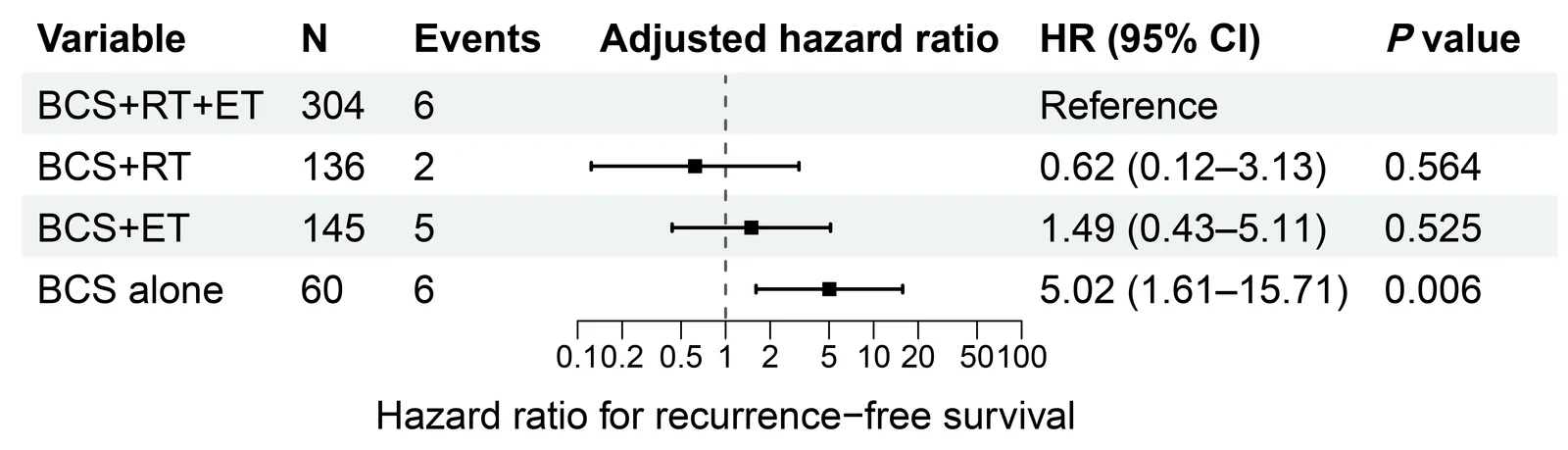
Multivariable Cox regression analyses of survival outcomes after breast-conserving surgery. BCS, breast-conserving surgery; RT, radiotherapy; ET, endocrine therapy; HR, hazard ratio; CI, confidence interval.

In the multivariable Cox proportional hazards model, with BCS + RT + ET as the reference group, BCS alone was associated with a higher hazard of RFS events (adjusted HR, 5.02; 95% CI, 1.61–15.71; *p* = 0.006). In contrast, no statistically significant differences in RFS were observed for BCS + RT (adjusted HR, 0.62; 95% CI, 0.12–3.13; *p* = 0.564) or BCS + ET (adjusted HR, 1.49; 95% CI, 0.43–5.11; *p* = 0.525) compared with BCS + RT + ET (Figure 3).

### 3.3. IPTW-Adjusted Comparison of Single and Dual Adjuvant Therapies

Because postoperative treatment allocation was determined by clinical decision-making rather than randomization, inverse probability of treatment weighting (IPTW) analysis was performed to improve comparability between patients receiving single-modality and dual-modality adjuvant therapy. Before weighting, several clinicopathological characteristics showed imbalance between treatment groups, particularly age, tumor size, ER expression, and PR expression. After IPTW adjustment, covariate balance was substantially improved, with absolute standardized mean differences (SMDs) below 0.10 for all evaluated covariates in both comparisons (Supplementary Figure S1; Supplementary Tables S1 and S2). The distributions of stabilized weights showed no evidence of extreme values, and the effective sample sizes remained 403.8 and 361.8 for the two pairwise comparisons, respectively (Supplementary Table S4). Visual inspection of the propensity-score distributions demonstrated adequate overlap and common support between treatment groups (Supplementary Figure S2). Adjustment for diagnosis year alone had little effect on the treatment-effect estimates. For BCS + ET versus BCS + RT + ET, the HR changed from 1.82 (95% CI, 0.49–6.82; *p* = 0.373) in the primary IPTW model to 1.94 (95% CI, 0.53–7.11; *p* = 0.318) after adjustment for diagnosis year. After additional center stratification, the HR increased to 2.56 (95% CI, 0.71–9.19; *p* = 0.151). For BCS + RT versus BCS + RT + ET, the corresponding HRs were 0.28 (95% CI, 0.06–1.44; *p* = 0.128), 0.29 (95% CI, 0.06–1.49; *p* = 0.140), and 0.80 (95% CI, 0.14–4.72; *p* = 0.810), respectively. Although none of these comparisons reached statistical significance, the changes in point estimates after center stratification and the wide confidence intervals indicate uncertainty in the magnitude of the estimated treatment effects (Supplementary Figure S3).

**Figure 4:**
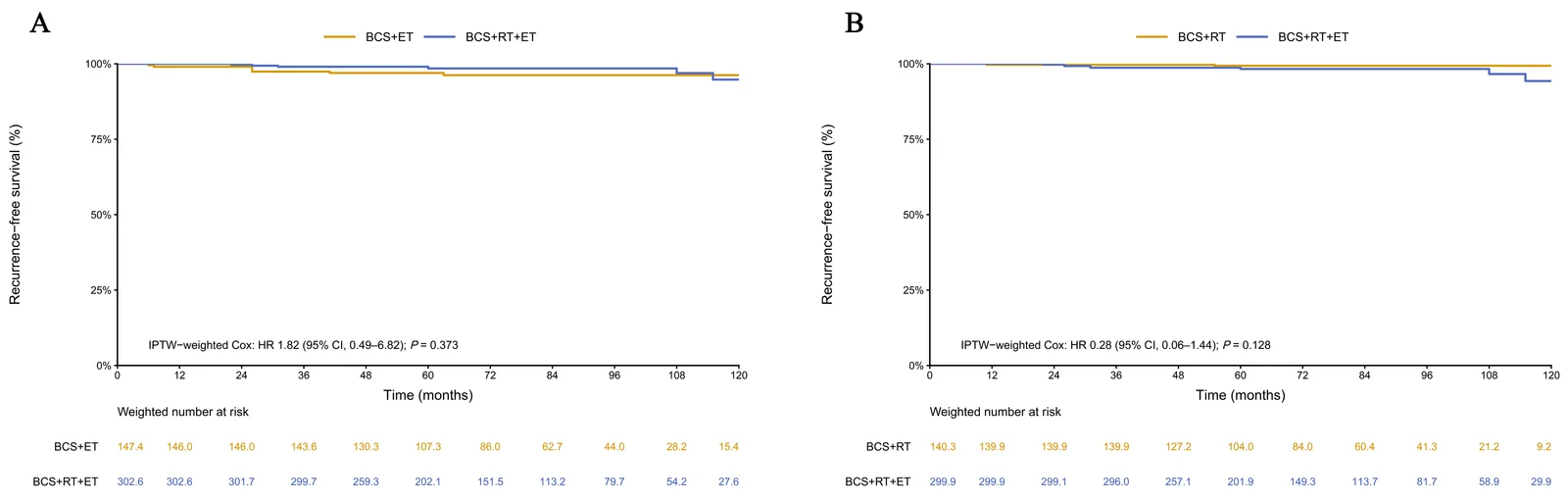
IPTW-adjusted survival outcomes according to adjuvant treatment strategies after breast-conserving surgery. (**A**) Recurrence-free survival (RFS) in patients receiving BCS + ET versus BCS + RT + ET. (**B**) RFS in patients receiving BCS + RT versus BCS + RT + ET. BCS, breast-conserving surgery; RT, radiotherapy; ET, endocrine therapy; IPTW, inverse probability of treatment weighting.

In the IPTW-adjusted comparison between BCS + ET and BCS + RT + ET, no statistically significant difference in recurrence-free survival was observed (HR, 1.82; 95% CI, 0.49–6.82; *p* = 0.373) (Figure 4A). Similarly, recurrence-free survival did not differ significantly between patients receiving BCS + RT and those receiving BCS + RT + ET after IPTW adjustment (HR, 0.28; 95% CI, 0.06–1.44; *p* = 0.128) (Figure 4B).

Overall, the IPTW-adjusted analyses yielded treatment-effect estimates broadly consistent with the primary multivariable Cox regression analysis. However, given the small number of RFS events and wide confidence intervals, these findings should be interpreted as imprecise and do not establish equivalence between single- and dual-modality treatment strategies.

## 4. Discussion

DCIS is a biologically heterogeneous disease, and the optimal selection of postoperative adjuvant therapy after BCS remains an important clinical challenge, particularly among patients with hormone receptor-positive disease. In the current era of increasing concerns regarding overdiagnosis and overtreatment of DCIS, we established the first large-scale real-world cohort study of Chinese patients with HR+ DCIS to comprehensively evaluate the comparative effectiveness of single-modality and combined dual-modality adjuvant therapies (RT + ET) following BCS.

In this multicenter cohort, the addition of a second adjuvant modality did not demonstrate a statistically significant improvement in RFS compared with single-modality therapy. Only 19 first RFS events occurred among the 645 patients, comprising 7 ipsilateral breast events (1 invasive recurrence and 6 DCIS recurrences), 7 contralateral breast events (2 invasive cancers and 5 DCIS events), 2 distant recurrences, and 3 deaths without recurrence; no regional recurrences were observed (Supplementary Table S3). With only 2–6 events in each treatment group, this sparse and clinically heterogeneous event distribution limits the precision of the treatment-effect estimates and the feasibility of reliable event-specific analyses. The wide confidence intervals in the primary IPTW comparisons (BCS + ET vs. BCS + RT + ET: HR, 1.82; 95% CI, 0.49–6.82; BCS + RT vs. BCS + RT + ET: HR, 0.28; 95% CI, 0.06–1.44) further illustrate this uncertainty and warrant cautious interpretation of the incremental benefit of combined RT and ET. The sensitivity analyses provide additional context for interpreting these estimates. Adjustment for diagnosis year alone had little influence on the results, whereas additional center stratification resulted in appreciable changes in the point estimates, particularly for BCS + RT versus BCS + RT + ET. These findings suggest that the estimated magnitude of treatment effects may be sensitive to institutional heterogeneity. Accordingly, the sensitivity analyses should be interpreted cautiously and do not establish the robustness of the treatment-effect estimates or equivalence between treatment strategies. Our results nevertheless provide real-world comparative data that may inform future studies designed to more precisely define the relative benefits of different adjuvant treatment strategies in HR+ DCIS.

Previous randomized trials and meta-analyses have established the effectiveness of radiotherapy (RT) and endocrine therapy (ET) in reducing subsequent breast events after breast-conserving surgery (BCS) for DCIS. The Early Breast Cancer Trialists’ Collaborative Group (EBCTCG) meta-analysis demonstrated that adjuvant RT following BCS approximately halved the risk of ipsilateral invasive and non-invasive local recurrence, supporting RT as an effective strategy for improving local control [11]. Similarly, the NSABP B-24 trial demonstrated that tamoxifen reduced subsequent breast cancer events, including ipsilateral and contralateral breast cancer events, among patients with ER-positive DCIS. The UK/ANZ DCIS trial further showed that tamoxifen reduced ipsilateral breast events after local excision of DCIS [12,13]. Consistent with these previous findings, patients receiving adjuvant therapy in our cohort experienced fewer RFS events than those undergoing BCS alone. However, this comparison should be interpreted cautiously because patients who did not receive RT or ET may differ systematically from those receiving adjuvant therapy, and reasons for treatment omission were not systematically captured in this retrospective multicenter cohort. Therefore, residual confounding by indication cannot be completely excluded. Beyond the comparison with BCS alone, our study addressed the unresolved question of whether combined RT and ET provides additional clinical benefit compared with single-modality therapy among patients with HR+ DCIS undergoing BCS. In both multivariable Cox regression and IPTW-adjusted analyses, no statistically significant difference in RFS was detected between dual-modality therapy and either single-modality strategy. However, given the limited number of events and wide confidence intervals, these findings should be interpreted cautiously. Compared with previous studies that primarily evaluated the efficacy of individual treatment modalities versus observation or placebo-controlled strategies, our multicenter real-world cohort provides complementary evidence by directly comparing single- and dual-modality adjuvant strategies within a contemporary HR+ DCIS population, thereby informing future individualized treatment selection and de-escalation approaches.

The clinical value of adding endocrine therapy to radiotherapy in DCIS remains an area of ongoing debate. In the NSABP B-24 trial, the addition of tamoxifen after breast-conserving surgery and radiotherapy reduced subsequent breast cancer events among patients with ER-positive DCIS, supporting the potential benefit of combined endocrine and radiation therapy in selected patients [12]. However, subsequent studies have suggested that the magnitude of this benefit may vary according to patient characteristics and treatment context. Differences in study populations, endpoint definitions, follow-up duration, treatment adherence, and the number of observed events may contribute to the heterogeneous findings across studies.

The long-term follow-up results of the UK/ANZ DCIS trial provided important insights into the potential interaction between tamoxifen and radiotherapy in DCIS. In that study, tamoxifen was associated with a reduction in ipsilateral breast events among patients who did not receive radiotherapy (RT) (HR, 0.77; 95% CI, 0.59–0.98; *p* = 0.04), whereas no statistically significant benefit was observed among patients who received RT (HR, 0.93; 95% CI, 0.50–1.75; *p* = 0.80) [13]. Similarly, de Sousa et al. reported that the addition of endocrine therapy was not associated with a statistically significant improvement in recurrence outcomes among HR+ DCIS patients who had already undergone BCS combined with RT [14]. Consistent with these observations, our study further demonstrated that dual-modality therapy did not provide a statistically significant improvement in RFS compared with either single-modality strategy after adjustment using multivariable Cox regression and IPTW analyses. Together, these findings suggest that the incremental benefit of adding endocrine therapy to radiotherapy may vary across different clinical settings. However, estimates of any additional benefit in our cohort remain imprecise because of the limited number of RFS events and wide confidence intervals. These observations should therefore be considered hypothesis-generating and warrant confirmation in larger, adequately powered prospective studies.

In exploratory comparisons, recurrence rates were numerically lower among patients receiving RT than among those receiving ET as a single-modality adjuvant treatment, although this difference did not reach statistical significance. This finding should be interpreted cautiously given the limited number of outcome events and the observational nature of our study. The potential difference between these two treatment approaches may be related to their distinct therapeutic mechanisms and clinical implementation. Radiotherapy exerts local cytotoxic effects by inducing DNA damage in residual microscopic disease within the breast, thereby improving local control [15]. In contrast, endocrine therapy suppresses the proliferation of hormone-dependent tumor cells through estrogen receptor blockade or estrogen deprivation. In real-world settings, the effectiveness of long-term ET may also be influenced by treatment adherence and tolerance, as therapy-related adverse effects can affect treatment persistence [16,17]. However, adherence data and treatment-related toxicities were not captured in the present cohort, and their contribution to the observed recurrence patterns remains uncertain. Further studies incorporating larger cohorts, longer follow-up, and comprehensive assessment of treatment adherence and patient-reported outcomes are warranted to better define the relative benefits of RT and ET in selected patients with HR+ DCIS.

In recent years, multiple prospective clinical trials have focused on evaluating de-escalation strategies for selected patients with low-risk DCIS, aiming to balance oncological safety with reduction of treatment burden. These efforts encompass different aspects of DCIS management, including surgery, radiotherapy, and endocrine therapy. At the surgical level, the COMET, LORIS, and LORD trials are evaluating active surveillance as an alternative approach to immediate surgical excision in carefully selected low-risk DCIS populations [18,19,20]. In the field of radiotherapy, several ongoing de-escalation trials (e.g., NCT04797299 and NCT03878342) are investigating whether selected patients with biologically favorable DCIS may achieve acceptable outcomes with omission or reduction of adjuvant RT after BCS. In addition, the TBCC-ARO DCIS trial represents an ongoing effort to evaluate whether low-dose tamoxifen may provide a less intensive endocrine strategy for patients with estrogen receptor-positive DCIS [8]. Together with these ongoing prospective efforts, our findings provide real-world comparative data that may help inform future investigations of individualized treatment intensity. However, given the limited statistical power of the present study, these findings should be interpreted cautiously and should not be considered sufficient evidence to support routine treatment de-escalation.

Several limitations should be acknowledged. First, only 19 RFS events occurred among the 645 patients, resulting in limited statistical power, wide confidence intervals, and potential model instability, thereby limiting the precision of treatment-effect estimates. Accordingly, the non-significant findings should be interpreted cautiously and do not establish equivalence between treatment strategies. In addition, RFS was a composite endpoint encompassing clinically heterogeneous events, which may obscure treatment-specific effects on individual event types. Although the median follow-up was 74.0 months, a longer observation period may be needed to more fully capture late recurrences in DCIS. Second, treatment allocation was based on clinical decision-making rather than randomization. Although IPTW substantially improved balance across measured baseline covariates, propensity score-based weighting cannot eliminate confounding from unmeasured or incompletely measured factors. Residual confounding may therefore remain, particularly for comparisons involving the BCS-alone group, because reasons for omission of radiotherapy or endocrine therapy were not systematically available in the database. Detailed clinical and treatment-related variables, including radiotherapy parameters, comedo necrosis status, comprehensive imaging characteristics, and treatment initiation timing, were not consistently available either. In addition, the appreciable changes in point estimates after center stratification suggest that institutional heterogeneity may influence the estimated treatment effects. Restriction to patients with negative surgical margins (>2 mm) and the high proportion of mass-related presentations may limit the generalizability of our findings to patients with close or positive margins and to predominantly screening-detected DCIS populations. Third, treatment-related toxicities, endocrine therapy adherence, and patient-reported quality-of-life outcomes were not systematically collected. Endocrine therapy was therefore analyzed as a binary exposure because detailed information regarding treatment duration and adherence was unavailable for a substantial proportion of patients. Future prospective studies incorporating larger cohorts, standardized assessment of treatment exposure, patient-reported outcomes, and validated risk stratification approaches are warranted to further refine individualized adjuvant treatment strategies for patients with HR+ DCIS.

## 5. Conclusions

Among patients with HR+ DCIS undergoing BCS, no statistically significant difference in RFS was observed between dual-modality and single-modality adjuvant therapy. However, the limited number of RFS events and wide confidence intervals indicate substantial uncertainty in the treatment-effect estimates, and the absence of statistical significance should not be interpreted as evidence of equivalence between treatment strategies. Therefore, the magnitude of the incremental benefit of combined RT and ET remains uncertain. Larger prospective studies with adequate statistical power are warranted to more precisely define the comparative effectiveness of these treatment strategies.

## Figures and Tables

**Table 1:** Patient and clinicopathological characteristics of the study cohort.

	All Treatments	BCS Alone	BCS + RT	BCS + ET	BCS + RT + ET	*p* Value
**Total**	645	60 (9.3)	136 (21.1)	145 (22.5)	304 (47.1)	
**Tumor size**						
≤20 mm	462 (71.6)	39 (65.0)	88 (64.7)	98 (67.6)	237 (78.0)	0.009
>20 mm	183 (28.4)	21 (35.0)	48 (35.3)	47 (32.4)	67 (22.0)	
**Histological grade**						
I–II	497 (77.1)	51 (85.0)	99 (72.8)	110 (75.9)	237 (78.0)	0.284
III	148 (22.9)	9 (15.0)	37 (27.2)	35 (24.1)	67 (22.0)	
**Ki-67**						
≥15%	240 (37.2)	23 (38.3)	61 (44.9)	48 (33.1)	108 (35.5)	0.183
<15%	405 (62.8)	37 (61.7)	75 (55.1)	97 (66.9)	196 (64.5)	
**Age**						
≤50	382 (59.2)	32 (53.3)	88 (64.7)	74 (51.0)	188 (61.8)	<0.001
51–70	214 (33.2)	20 (33.3)	45 (33.1)	44 (30.3)	105 (34.5)	
>70	49 (7.6)	8 (13.3)	3 (2.2)	27 (18.6)	11 (3.6)	
**Multifocal**						
Yes	72 (11.2)	3 (5.0)	11 (8.1)	24 (16.6)	34 (11.2)	0.058
No	573 (88.8)	57 (95.0)	125 (91.9)	121 (83.4)	270 (88.8)	
**Manifestation**						
Mass	515 (79.8)	50 (83.3)	118 (86.8)	114 (78.6)	233 (76.6)	0.087
Non-mass	130 (20.2)	10 (16.7)	18 (13.2)	31 (21.4)	71 (23.4)	
**ER status**						
≤10%	73 (11.3)	13 (21.7)	36 (26.5)	12 (8.3)	12 (3.9)	<0.001
>10%	572 (88.7)	47 (78.3)	100 (73.5)	133 (91.7)	292 (96.1)	
**PR status**						
≤10%	167 (25.9)	23 (38.3)	46 (33.8)	32 (22.1)	66 (21.7)	0.004
>10%	478 (74.1)	37 (61.7)	90 (66.2)	113 (77.9)	238 (78.3)	
**Menopausal status**						
Premenopausal	393 (60.9)	34 (56.7)	90 (66.2)	73 (50.3)	196 (64.5)	0.015
Postmenopausal	252 (39.1)	26 (43.3)	46 (33.8)	72 (49.7)	108 (35.5)	

Abbreviations: BCS, breast-conserving surgery; RT, radiotherapy; ET, endocrine therapy; ER, estrogen receptor; PR, progesterone receptor.

## Data Availability

The data presented in this study are available on request from the corresponding author. The data are not publicly available owing to restrictions relating to patient privacy.

## Supplementary Materials

The following supporting information can be downloaded at https://www.mdpi.com/article/10.3390/jcm15218729/s1. Figure S1. Covariate balance before and after inverse probability of treatment weighting. (A) BCS + ET versus BCS + RT + ET. (B) BCS + RT versus BCS + RT + ET. The dashed vertical line indicates an absolute SMD threshold of 0.10, representing adequate covariate balance after IPTW. BCS, breast-conserving surgery; RT, radiotherapy; ET, endocrine therapy; IPTW, inverse probability of treatment weighting; SMD, standardized mean difference; ER, estrogen receptor; PR, progesterone receptor. Figure S2. Propensity-score distributions before and after inverse probability of treatment weighting. (A) BCS + ET versus BCS + RT + ET. (B) BCS + RT versus BCS + RT + ET. Propensity-score distributions are shown before weighting and after application of stabilized inverse probability of treatment weights. Visual inspection suggested adequate overlap and common support between the treatment groups. BCS, breast-conserving surgery; RT, radiotherapy; ET, endocrine therapy; IPTW, inverse probability of treatment weighting. Figure S3. Sensitivity analyses of IPTW-weighted Cox regression models. Sensitivity analyses were performed using IPTW-weighted Cox regression models with additional adjustment for diagnosis year alone and diagnosis year-plus-center stratification. (A) BCS + ET versus BCS + RT + ET. (B) BCS + RT versus BCS + RT + ET. Hazard ratios (HRs) with 95% confidence intervals (CIs) are shown. BCS, breast-conserving surgery; RT, radiotherapy; ET, endocrine therapy; IPTW, inverse probability of treatment weighting. Table S1. Baseline characteristics before and after inverse probability of treatment weighting in patients receiving BCS + ET versus BCS + RT + ET. Table S2. Baseline characteristics before and after inverse probability of treatment weighting in patients receiving BCS + RT versus BCS + RT + ET. Table S3. Distribution of first RFS events according to adjuvant treatment strategy. Table S4. Distribution of stabilized inverse probability of treatment weights and effective sample size after IPTW. Table S5. Distribution of eligible patients according to participating centers.

## References

[1] Narod S.A., Iqbal J., Giannakeas V., Sopik V., Sun P. (2015). Breast Cancer Mortality After a Diagnosis of Ductal Carcinoma In Situ. JAMA Oncol..

[2] Poli E.C., Dong W., Shaitelman S.F., Tamirisa N., Shen Y., Bedrosian I. (2024). Survival outcomes after omission of surgery for ductal carcinoma in situ. npj Breast Cancer.

[3] Bleyer A., Welch H.G. (2012). Effect of three decades of screening mammography on breast-cancer incidence. N. Engl. J. Med..

[4] Delaloge S., Khan S.A., Wesseling J., Whelan T. (2024). Ductal carcinoma in situ of the breast: Finding the balance between overtreatment and undertreatment. Lancet.

[5] Wang J., Li B., Luo M., Huang J., Zhang K., Zheng S., Zhang S., Zhou J. (2024). Progression from ductal carcinoma in situ to invasive breast cancer: Molecular features and clinical significance. Signal Transduct. Target Ther..

[6] Wapnir I.L., Dignam J.J., Fisher B., Mamounas E.P., Anderson S.J., Julian T.B., Land S.R., Margolese R.G., Swain S.M., Costantino J.P. (2011). Long-term outcomes of invasive ipsilateral breast tumor recurrences after lumpectomy in NSABP B-17 and B-24 randomized clinical trials for DCIS. J. Natl. Cancer Inst..

[7] Hwang E.S., Hyslop T., Lynch T., Ryser M.D., Weiss A., Wolf A., Norris K., Witten M., Grimm L., Schnitt S. (2025). Active Monitoring With or Without Endocrine Therapy for Low-Risk Ductal Carcinoma In Situ: The COMET Randomized Clinical Trial. JAMA.

[8] Kuo S.-H., Tseng L.-M., Chen S.-T., Sagara Y., Chang Y.-C., Yeh H.-T., Kuo Y.-L., Hung C.-C., Lu T.-P., Lee Y.-H. (2023). Radiotherapy versus low-dose tamoxifen following breast-conserving surgery for low-risk and estrogen receptor-positive breast ductal carcinoma in situ: An international open-label randomized non-inferiority trial (TBCC-ARO DCIS Trial). BMC Cancer.

[9] Lupinacci K., Hamed A., Truong E., Goktepe B., Soran A. (2025). De-escalation of Endocrine Therapy in Patients with Ductal Carcinoma In Situ After Mastectomy. Ann. Surg. Oncol..

[10] Wang Y., Peng D., Zhou X., Hu W., Li F. (2024). Treatments and Prognosis of the Breast Ductal Carcinoma In Situ. Clin. Breast Cancer.

[11] De Laurentiis M., Del Mastro L., Early Breast Cancer Trialists’ Collaborative Group (EBCTCG) (2010). Overview of the randomized trials of radiotherapy in ductal carcinoma in situ of the breast. J. Natl. Cancer Inst. Monogr..

[12] Allred D.C., Anderson S.J., Paik S., Wickerham D.L., Nagtegaal I.D., Swain S.M., Mamounas E.P., Julian T.B., Geyer C.E., Costantino J.P. (2012). Adjuvant tamoxifen reduces subsequent breast cancer in women with estrogen receptor-positive ductal carcinoma in situ: A study based on NSABP protocol B-24. J. Clin. Oncol..

[13] Cuzick J., Sestak I., Pinder S.E., Ellis I.O., Forsyth S., Bundred N.J., Forbes J.F., Bishop H., Fentiman I.S., George W.D. (2011). Effect of tamoxifen and radiotherapy in women with locally excised ductal carcinoma in situ: Long-term results from the UK/ANZ DCIS trial. Lancet Oncol..

[14] de Sousa C.F.P.M., Pereira A.A.L., Arruda G.V., Gouveia A.G., Hanna S.A., Cruz M.R.d.S., dos Anjos C.H., Bevilacqua J.L.B., Filho P.A., de Moraes F.Y. (2023). Real-World Evidence on the Use of Endocrine Therapy for Ductal Carcinoma In Situ in Patients Treated With Breast-Conserving Surgery Followed by Postoperative Radiation Therapy: A Brazilian Retrospective Cohort Study. Clin. Breast Cancer.

[15] Morgan M.A., Lawrence T.S. (2015). Molecular Pathways: Overcoming Radiation Resistance by Targeting DNA Damage Response Pathways. Clin. Cancer Res..

[16] Clusan L., Ferrière F., Flouriot G., Pakdel F. (2023). A Basic Review on Estrogen Receptor Signaling Pathways in Breast Cancer. Int. J. Mol. Sci..

[17] Fleming L., Agnew S., Peddie N., Crawford M., Dixon D., MacPherson I. (2022). The impact of medication side effects on adherence and persistence to hormone therapy in breast cancer survivors: A quantitative systematic review. Breast.

[18] Hwang E.S., Hyslop T., Lynch T., Frank E., Pinto D., Basila D., Collyar D., Bennett A., Kaplan C., Rosenberg S. (2019). The COMET (Comparison of Operative versus Monitoring and Endocrine Therapy) trial: A phase III randomised controlled clinical trial for low-risk ductal carcinoma in situ (DCIS). BMJ Open.

[19] Francis A., Thomas J., Fallowfield L., Wallis M., Bartlett J.M., Brookes C., Roberts T., Pirrie S., Gaunt C., Young J. (2015). Addressing overtreatment of screen detected DCIS; the LORIS trial. Eur. J. Cancer.

[20] Schmitz R.S., Engelhardt E.G., Gerritsma M.A., Sondermeijer C.M., Verschuur E., Houtzager J., Griffioen R., Retèl V., Bijker N., Mann R.M. (2023). Active surveillance versus treatment in low-risk DCIS: Women’s preferences in the LORD-trial. Eur. J. Cancer.

