# Supplementary material for: Comparative Effectiveness of Single Versus Dual Adjuvant Therapy After Breast-Conserving Surgery in Hormone Receptor-Positive Ductal Carcinoma In Situ: A Multicenter Retrospective Cohort Study
Source: J Clin Med. 2026 Nov 9;15(21):8729. doi: 10.3390/jcm15218729 (PMC13764119; doi:10.3390/jcm15218729)
Supplement: Supplementary file 1 [file jcm-15-08729-s001.zip › Supplementary Table S1.pdf]

Supplementary Table S1. Baseline characteristics before and after inverse probability of treatment weighting in patients receiving BCS+ET versus BCS+RT+ET

Variable	Before IPTW				After IPTW			
	Total	BCS+ET	BCS+RT+ET	SMD	Total	BCS+ET	BCS+RT+ET	SMD
N (weight)	449	145	304		450	147.4	302.6	
Age (years, median [IQR])	48 [42, 59]	50 [44, 63]	47 [42, 56]	0.413	48 [42, 59]	48 [42, 56]	49 [43, 60]	0.028
Tumor size (mm, median [IQR])	13 [10, 21]	15 [10, 25]	13 [10, 20]	0.235	13 [10, 21]	12 [9, 22]	13 [10, 20]	0.010
Ki-67 labeling index (%), median [IQR]	10 [5, 20]	10 [5, 20]	10 [5, 20]	0.022	10 [5, 20]	10 [3, 20]	10 [5, 20]	0.003
ER expression (%), median [IQR]	90 [70, 95]	90 [60, 95]	90 [75, 95]	0.321	90 [70, 95]	90 [70, 95]	90 [70, 95]	0.021
PR expression (%), median [IQR]	70 [15, 90]	55 [15, 90]	75 [20, 90]	0.210	70 [15, 90]	70 [15, 90]	70 [15, 90]	0.032
Histological grade, n (%)				0.053				0.024
Grade I	116 (25.8)	36 (24.8)	80 (26.3)		114.4 (25.4)	37.3 (25.3)	77.1 (25.5)	
Grade II	231 (51.5)	74 (51.0)	157 (51.7)		229.7 (51.1)	74.4 (50.5)	155.3 (51.3)	
Grade III	102 (22.7)	35 (24.2)	67 (22.0)		105.9 (23.5)	35.7 (24.2)	70.2 (23.2)	
Multifocality, n (%)				0.156				0.028
Yes	58 (12.9)	24 (16.6)	34 (11.2)		55.9 (12.4)	17.4 (11.8)	38.5 (12.7)	
No	391 (87.1)	121 (83.4)	270 (88.8)		394.2 (87.6)	130.0 (88.2)	264.2 (87.3)	

Abbreviations: IPTW, inverse probability of treatment weighting; SMD, standardized mean difference; BCS, breast-conserving surgery; RT, radiotherapy; ET, endocrine therapy; ER, estrogen receptor; PR, progesterone receptor; IQR, inter-quartile range.
