# Supplementary material for: Comparative Effectiveness of Single Versus Dual Adjuvant Therapy After Breast-Conserving Surgery in Hormone Receptor-Positive Ductal Carcinoma In Situ: A Multicenter Retrospective Cohort Study
Source: J Clin Med. 2026 Nov 9;15(21):8729. doi: 10.3390/jcm15218729 (PMC13764119; doi:10.3390/jcm15218729)
Supplement: Supplementary file 1 [file jcm-15-08729-s001.zip › Supplementary Table S2.pdf]

Supplementary Table S2. Baseline characteristics before and after inverse probability of treatment weighting in patients receiving BCS+RT versus BCS+RT+ET

Variable	Before IPTW				After IPTW			
	Total	BCS+RT	BCS+RT+ET	SMD	Total	BCS+RT	BCS+RT+ET	SMD
N (weight)	440	136	304		440.3	140.3	299.9	
Age (years, median [IQR])	48 [42, 56]	48 [41, 54]	47 [42, 56]	0.097	48 [42, 55]	49 [42, 56]	47 [42, 55]	0.016
Tumor size (mm, median [IQR])	14 [10, 22]	15 [10, 26]	13 [10, 20]	0.338	13 [10, 20]	12 [10, 20]	15 [10, 20]	0.029
Ki-67 labeling index (%), median [IQR]	10 [5, 20]	10 [5, 20]	10 [5, 20]	0.164	10 [5, 20]	10 [5, 20]	10 [5, 20]	0.002
ER expression (%), median [IQR]	90 [60, 95]	65 [10, 90]	90 [75, 95]	0.874	90 [60, 95]	90 [70, 95]	90 [60, 95]	0.015
PR expression (%), median [IQR]	70 [10, 90]	30 [5, 80]	75 [20, 90]	0.485	70 [10, 90]	70 [15, 90]	70 [10, 90]	0.001
Histological grade, n (%)				0.123				0.045
Grade I	112 (25.5)	32 (23.5)	80 (26.3)		111.2 (25.2)	33.9 (24.1)	77.4 (25.8)	
Grade II	224 (50.9)	67 (49.3)	157 (51.7)		221.3 (50.3)	70.7 (50.4)	150.6 (50.2)	
Grade III	104 (23.6)	37 (27.2)	67 (22.0)		107.7 (24.5)	35.8 (25.5)	71.9 (24.0)	
Multifocality, n (%)				0.105				0.014
Yes	45 (10.2)	11 (8.1)	34 (11.2)		44.7 (10.2)	13.8 (9.9)	30.9 (10.3)	
No	395 (89.8)	125 (91.9)	270 (88.8)		395.6 (89.8)	126.5 (90.1)	269.1 (89.7)	

Abbreviations: IPTW, inverse probability of treatment weighting; SMD, standardized mean difference; BCS, breast-conserving surgery; RT, radiotherapy; ET, endocrine therapy; ER, estrogen receptor; PR, progesterone receptor; IQR, inter-quartile range.
