# Supplementary material for: Comparative Effectiveness of Single Versus Dual Adjuvant Therapy After Breast-Conserving Surgery in Hormone Receptor-Positive Ductal Carcinoma In Situ: A Multicenter Retrospective Cohort Study
Source: J Clin Med. 2026 Nov 9;15(21):8729. doi: 10.3390/jcm15218729 (PMC13764119; doi:10.3390/jcm15218729)
Supplement: Supplementary file 1 [file jcm-15-08729-s001.zip › Supplementary Table S3.pdf]

Supplementary Table S3. Distribution of first RFS events according to adjuvant treatment strategy

	BCS alone<br>N=60	BCS+RT<br>N=136	BCS+ET<br>N=145	BCS+RT+ET<br>N=304	Total<br>N=645
**RFS events**	6(10.0%)	2(1.5%)	5(3.4%)	6(2.0%)	19(2.9%)
**Ipsilateral breast events**					
Invasive	0(0.0%)	0(0.0%)	0(0.0%)	1(0.3%)	1(0.2%)
DCIS	3(5.0%)	1(0.7%)	0(0.0%)	2(0.7%)	6(0.9%)
**Contralateral breast events**					
Invasive	0(0.0%)	0(0.0%)	1(0.7%)	1(0.3%)	2(0.3%)
DCIS	0(0.0%)	1(0.7%)	2(1.4%)	2(0.7%)	5(0.8%)
**Regional recurrence**	0(0.0%)	0(0.0%)	0(0.0%)	0(0.0%)	0(0.0%)
**Distant recurrence**					
Liver	1(1.7%)	0(0.0%)	0(0.0%)	0(0.0%)	1(0.2%)
Lung	1(1.7%)	0(0.0%)	0(0.0%)	0(0.0%)	1(0.2%)
**Death without recurrence**	1(1.7%)	0(0.0%)	2(1.4%)	0(0.0%)	3(0.5%)

Abbreviations: BCS, breast-conserving surgery; RT, radiotherapy; ET, endocrine therapy; DCIS, ductal carcinoma in situ; RFS, recurrence-free survival.
