# Supplementary material for: Comparative Effectiveness of Single Versus Dual Adjuvant Therapy After Breast-Conserving Surgery in Hormone Receptor-Positive Ductal Carcinoma In Situ: A Multicenter Retrospective Cohort Study
Source: J Clin Med. 2026 Nov 9;15(21):8729. doi: 10.3390/jcm15218729 (PMC13764119; doi:10.3390/jcm15218729)
Supplement: Supplementary file 1 [file jcm-15-08729-s001.zip › Supplementary Table S4.pdf]

Supplementary Table S4. Distribution of stabilized inverse probability of treatment weights and effective sample size after IPTW

Comparison	Original N	ESS	Mean weight	SD weight	Min weight	P1	Median	P99	Max weight
BCS+ET vs BCS+RT+ET	449	403.8	1.002	0.336	0.372	0.465	0.930	2.251	3.451
BCS+RT vs BCS+RT+ET	440	361.8	1.001	0.466	0.351	0.379	0.858	2.628	2.988

Abbreviations: IPTW, inverse probability of treatment weighting; ESS, effective sample size; SD, standard deviation; P1 and P99, 1st and 99th percentiles of the stabilized weights.
