# Supplementary material for: Comparative Effectiveness of Single Versus Dual Adjuvant Therapy After Breast-Conserving Surgery in Hormone Receptor-Positive Ductal Carcinoma In Situ: A Multicenter Retrospective Cohort Study
Source: J Clin Med. 2026 Nov 9;15(21):8729. doi: 10.3390/jcm15218729 (PMC13764119; doi:10.3390/jcm15218729)
Supplement: Supplementary file 1 [file jcm-15-08729-s001.zip › Supplementary Table S5.pdf]

Supplementary Table S5. Distribution of eligible patients according to participating centers

Center ID	Number of patients
AA	201
AB	6
AC	9
AD	3
AE	29
AF	1
AG	16
AH	5
AI	4
AJ	16
AK	8
AL	5
AM	6
AN	1
AO	4
AP	9
AQ	31
AR	13
AS	12
AT	6
AW	8
AX	9
AY	2
AZ	4
BA	25
BB	3
BC	23
BD	45
BE	6
BF	6
BH	87
BI	2
BJ	33
BL	2
BN	2
G	2
R	1
Total	645

Center identifiers were anonymized according to the institutional data-sharing framework. Only centers contributing eligible patients to the present analysis are listed, and center codes are presented instead of institutional names to preserve data confidentiality.
